# *Porphyromonas gingivalis* Vaccine: Antigens and Mucosal Adjuvants

**DOI:** 10.3390/vaccines12060619

**Published:** 2024-06-04

**Authors:** Shuo Wang, Tong Yan, Bingtao Zhang, Yixiang Chen, Zhitao Li

**Affiliations:** 1 College of Basic Medicine and Forensic Medicine, Henan University of Science and Technology, Luoyang 471003, China; 210321201183@stu.haust.edu.cn (S.W.); 211518010731@stu.haust.edu.cn (T.Y.); 211518010131@stu.haust.edu.cn (B.Z.); chenyixiang@lypt.edu.cn (Y.C.); 2 Henan Engineering Research Center for Key Immunological Biomaterials, Luoyang Polytechnic, Luoyang 471000, China

**Keywords:** *Porphyromonas gingivalis*, vaccine, antigen, immune adjuvant, immune pathway

## Abstract

*Porphyromonas gingivalis* (Pg), a Gram-negative anaerobic bacterium found in dental plaque biofilm within periodontal pockets, is the primary pathogenic microorganism responsible for chronic periodontitis. Infection by Pg significantly impacts the development and progression of various diseases, underscoring the importance of eliminating this bacterium for effective clinical treatment. While antibiotics are commonly used to combat Pg, the rise of antibiotic resistance poses a challenge to complete eradication. Thus, the prevention of Pg infection is paramount. Research suggests that surface antigens of Pg, such as fimbriae, outer membrane proteins, and gingipains, can potentially be utilized as vaccine antigens to trigger protective immune responses. This article overviews these antigens, discusses advancements in mucosal adjuvants (including immunostimulant adjuvants and vaccine-delivery adjuvants), and their application in Pg vaccine development. Furthermore, the review examines the advantages and disadvantages of different immune pathways and common routes of Pg vaccine immunization. By summarizing the current landscape of Pg vaccines, addressing existing challenges, and highlighting the potential of mucosal vaccines, this review offers new insights for the advancement and clinical implementation of Pg vaccines.

## 1. Introduction

*Porphyromonas gingivalis* (Pg) is a Gram-negative anaerobic bacterium found in the biofilm within the periodontal pocket. Pathogenic factors associated with Pg can be categorized into two groups. The first group includes self-pathogenic components such as fimbriae, flagella, and outer membrane proteins. The second group comprises pathogenic substances that Pg secreted, such as capsule polysaccharides, lipopolysaccharides, and gingipains [1,2]. These virulence factors allow Pg to adhere and colonize within the gingival sulcus, contributing to the development of periodontal diseases [1]. Moreover, Pg is linked to various diseases like diabetes [3,4], atherosclerosis [5], and different types of cancers, including oral [6], esophageal [7], pancreatic cancer [8], etc., significantly impacting human health.

Epidemiological investigations have shown that the prevalence of Pg infection increases with age [9]. Prolonged use of antibiotics can result in Pg developing resistance [10,11], posing challenges for eradication, as antibiotics are not the most effective treatment for Pg infections. Consequently, there is an increasing emphasis on controlling and preventing Pg infections. In recent years, vaccine development has evolved from traditional inactivated, attenuated, and subunit vaccines to modern DNA and RNA vaccines. These technological advancements play a critical role in developing vaccines that can effectively prevent Pg infections. As research has progressed, Pg vaccines have diversified in terms of their types and immunization methods. Given that Pg primarily colonizes the oral cavity, many studies focus on mucosal immune pathways [12,13,14], highlighting mucosal immunity as a promising approach for preventing Pg infections. This review delves into the three main factors (antigens, adjuvants, and immunization routes) involved in developing Pg vaccines, outlining the challenges encountered in research. Additionally, potential solutions are proposed, and future trends in Pg vaccine development are predicted.

## 2. Antigens Associated with Pg Vaccine

**Figure 1:**
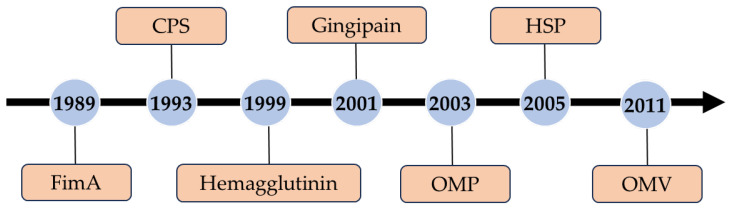
Timeline of Pg antigen discovery.

In the process of designing Pg vaccines, the selection of appropriate antigens is crucial. By choosing suitable antigens, the host’s immune system can be stimulated to produce specific antibodies and cellular immune responses, leading to immune protection against Pg. Numerous antigens of Pg have been the subject of research for many years, including surface proteins, structural proteins, polysaccharides, and outer membrane proteins (OMPs) (Figure 1). Surface proteins like fimbriae (FimA) and gingipain [15,16], which have been extensively researched, exhibit immunogenicity and adhesive capabilities, making them promising antigen candidates. Furthermore, capsular polysaccharides [17,18] and OMPs [19,20,21,22] are viable options for antigens.

### 2.1. Fimbriae

Fimbriae are crucial virulence factors of Pg, facilitating adhesion to host tissues and co-polymerization with other oral bacteria. Pg typically expresses long fimbriae FimA and short fimbriae Mfa1 encoded by the *fimA* and *mfa1* genes [23,24]. FimA, a major subunit of long fimbriae, is classified into six types (I, I b, II, III, IV and V) based on nucleotide sequences [25]. Types II and IV are most commonly detected in patients with periodontal disease, while type I is prevalent in periodontally healthy individuals [26,27,28]. The fimbrial hairs of Pg enable the adhesion and colonization of oral tissues and are highly immunogenic [28]. Research indicates that FimA monoclonal antibody can inhibit Pg adhesion [29]. Nasal immunization of BALB/c mice with FimA and recombinant cholera toxin B subunit (CTB) elicited a robust immune response and reduced alveolar bone loss caused by Pg infection [30]. Shin et al. demonstrated that mice fed genetically modified potatoes containing FimA developed high antibody titers in serum and saliva, enhancing resistance to Pg infection [31].

### 2.2. Hemagglutinin

Hemagglutinin (HA) is a crucial substance for the growth of Pg, as it helps Pg to attach to the surface of red blood cells [32,33]. The gene *hagA* in Pg encodes a surface protein called hemagglutinin A (HagA). Yuzawa et al. [34] created a fusion protein called HagA-MBP by combining HagA and MBP. The sublingual immunization of BALB/c mice with this fusion protein resulted in high levels of IgG and IgA in the serum and elevated secretory IgA (sIgA) antibodies in saliva. Apart from HagA, Pg also has hemagglutinins B, C, D and E (HagB, C, D and E). HagB is highly immunogenic and can trigger an immune response to protect against Pg infections and alveolar bone loss [35,36,37]. HagB is a key immunogen for developing a Pg vaccine, as it can activate TLR4 to induce an immune response [38].

### 2.3. Gingipains

Gingipains, including arginine gingipain (Rgp) and lysine gingipain (Kgp), are enzymes with protease activity secreted by Pg. These enzymes play a crucial role in various biological processes such as bacterial adherence, colonization, nutrient acquisition, cytotoxicity, the modulation of the inflammatory response, invasive and immune escape, and other functions [39,40]. They can cleave host tissue proteins, contributing to the development of diseases like gingivitis and periodontitis [41,42]. RgpA comprises a prepeptide region, a catalytic region, and an agglutination region that enhances Pg adhesion to erythrocytes and may stimulate Pg-specific IgG production for immunoprotection. On the other hand, RgpB lacks the agglutination region, suggesting that RgpA could be a more promising candidate antigen for vaccines [43,44]. Studies by Asaf Wilensky et al. [45] revealed the presence of specific IgG1 and IgG2a antibodies in serum following immunization with recombinant RgpA. Another study showed that immunization with the RgpA-Kgp protein adhesion complex induced a Th2 response in mice, effectively preventing alveolar bone loss and Pg infection [46,47].

### 2.4. Capsular Polysaccharide

Capsule polysaccharide (CPS) is a complex molecule on the surface of Pg, composed of various sugars and glycoconjugates. CPS can be categorized into seven serotypes, K1 to K7, and an unencapsulated strain, K-, with serotypes K5-K6 primarily associated with periodontitis [48,49,50,51]. Several studies have utilized CPS as an antigen to successfully prevent Pg-induced alveolar bone loss in mice through immunization [18,52]. Recently, Rocha et al. [17] developed a novel eCRM^®^ conjugate vaccine (Pg-CV) incorporating CPS, leading to higher IgG antibody levels in immunized mice than in the CPS group. This vaccine effectively protects against alveolar bone loss. Pg-CV represents a promising conjugate vaccine with the potential to target a broader spectrum of K serotypes in the future by utilizing different K antigens.

### 2.5. Outer Membrane Protein

Outer membrane protein (OMP) is crucial for Pg’s adhesion, nutrient acquisition, and protein secretion [53]. It has been observed in both the cell surface and extracellular membrane vesicles across various strains [19]. Numerous studies have explored the potential of OMP as a Pg vaccine, resulting in the production of protective IgG antibodies in all immunized mice, with higher levels in the group co-immunized with adjuvant [20,21,22]. Furthermore, OMP has been tested via the subcutaneous, oral, and intranasal immunization routes, leading to the production of specific IgG and sIgA antibodies against Pg infection [19,54,55]. Zhang et al. conducted sublingual immunization of mice with 40k-OMP, which significantly boosted serum IgG, IgA and salivary IgA antibody responses and notably reduced Pg-induced alveolar bone loss [56].

### 2.6. Heat-Shock Protein

Heat-shock proteins (HSP) of Pg, part of the molecular chaperone protein family, such as GroEL (HSP60) and DnaK (HSP70) [57], have conserved structures and functions that aid in interactions with other proteins for proper folding and assembly [58,59]. In individuals with periodontal disease, antibodies against HSP can be generated, indicating an immune response during infection, and the HSP sequences exhibit significant homology among periodontal bacteria [60,61,62]. Consequently, HSP is viewed as a potential target for periodontal disease vaccines. Studies by Chang et al. [12] and Lee et al. [62] showed that immunization with HSP60 resulted in specific antibody responses, reducing inflammation and bone loss caused by Pg and other periodontal pathogens. This suggests that HSP60 could be a promising candidate for use as a vaccine against periodontal disease induced by various bacterial infections.

### 2.7. Outer Membrane Vesicles

Outer membrane vesicles (OMV) are small vesicular structures secreted by Pg and enclosed by the outer membrane. These vesicles contain various components of the bacterial outer membrane, including OMP, lipopolysaccharides, gingipains and proteases [63,64]. Nakao et al. conducted intranasal immunization of BALB/c mice with OMV as the antigen and polyinosinic–polycytidylic acid (Poly (I:C)) as the mucosal adjuvant, resulting in high levels of secretory IgA in nasal wash and saliva, as well as serum IgG and IgA [65]. Furthermore, in a mouse model of oral infection, it was observed that mice immunized intranasally with OMV+Poly(I:C) exhibited a significant decrease in the number of Pg in the oral cavity compared to those immunized with Poly(I:C) alone, demonstrating the efficacy and safety of OMV immunization via the nasal route [66].

## 3. The Application of Mucosal Adjuvants in Pg Vaccine

**Figure 2:**
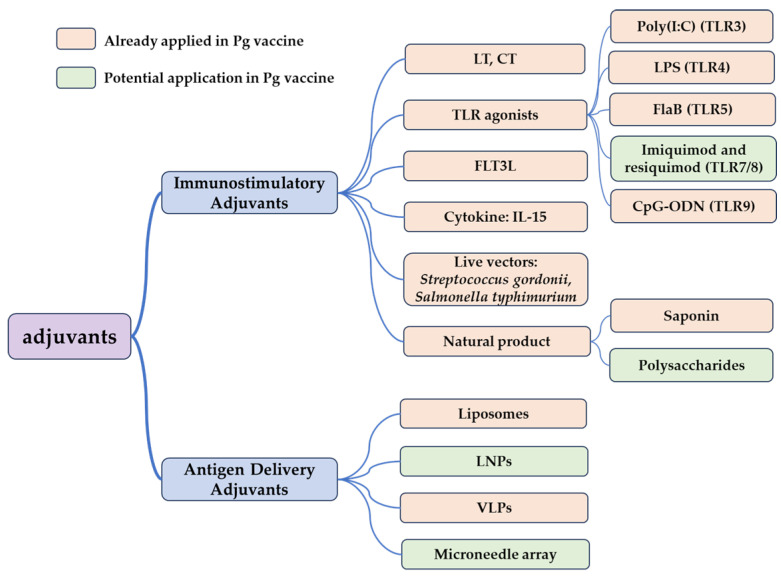
The application of adjuvants in Pg vaccine.

The primary function of adjuvants is to activate the innate immune response, enhance antigen presentation to the immune system, and prolong the presence of antigens in the body, continuously activating the immune system. Immunization effectiveness is often suboptimal when vaccines are administered alone; adjuvants are commonly used to achieve more robust and durable immune responses. Recent Pg vaccine research has focused on mucosal immunization, with traditional adjuvants like Freund’s adjuvant and aluminum adjuvants being less utilized. This review excludes traditional adjuvants, instead concentrating on commonly used adjuvants in mucosal vaccine systems (Table 1), broadly categorized as immunostimulant adjuvants and vaccine delivery adjuvants (Figure 2). Immunostimulant adjuvants, such as heat-labile enterotoxin (LT), cholera toxin (CT), TLR ligands, and cytokines, have been extensively studied in Pg vaccines, directly activating the innate immune system and promoting a robust immune response. Delivery systems like virus-like particles, polysaccharides, saponins, and microneedle arrays protect antigens from degradation after administration through various routes, ensuring sustained antigen release over an extended period [67,68]. However, these delivery systems are not widely used in Pg vaccines and may represent future development trends.

### 3.1. Immunostimulatory Adjuvants

#### 3.1.1. *Escherichia coli* Heat-Labile Enterotoxin and Cholera Toxin

*E. coli* heat-labile enterotoxin (LT) and cholera toxin (CT) are structurally similar toxins originating from *E.coli* and Vibrio cholerae, respectively. Both toxins consist of A and B subunits, with the A subunit being excluded from vaccines due to its toxicity. However, the non-toxic cholera toxin B subunit (CTB) can form a homopentameric structure that binds to epithelial cells, explicitly targeting ganglioside 1 (GM1) on their surfaces [69]. LT and CT, as well-established mucosal adjuvants, effectively boost antibody production in mucous membranes and serum, promoting long-lasting antigen memory. Widely utilized in vaccine research, these substances have paved the way for CTB [20,21,22], a safe derivative of CT [70], which is currently the sole subunit antigen in an approved mucosal vaccine [69]. Notably, Kim et al. [71] demonstrated that it is possible to enhance the response of B cells to oral immunization with CTB-FimA fusion proteins, potentially enhancing the efficacy of mucosal vaccines against periodontal disease. Furthermore, intranasal immunization of mice with LT and CTB combined with Kgp-rHArep resulted in enhanced Th1- and Th2-type immune responses, comparable to the effects of LT and MPL [72]. This suggests that combining CTB with antigens can significantly improve systemic and mucosal immune responses, underscoring their potential in vaccine development [72]. Recent studies have highlighted the safety and efficacy of *E.coli* double mutant heat-labile toxin (dmLT) as an oral vaccine adjuvant, showing promising results in clinical trials across various regions [73,74].

#### 3.1.2. Toll-like Receptor Agonists

Toll-like receptors (TLRs) are protein receptors found on cell surfaces or within cells that identify and bind to specific microbial molecules, triggering an antigen-specific immune response [75]. TLR agonists, which mimic pathogen molecular structures, activate immune cells and enhance host immune responses when they bind to TLRs. These agonists serve as both traditional injectable adjuvants and mucosal adjuvants in the oral, nasal, and intestinal mucosal immune systems. Examples of TLR agonists include double-stranded RNA (TLR3 agonist), bacterial lipopolysaccharide (TLR4 agonist), bacterial flagellin (TLR5 agonist), and CpG oligodeoxynucleotides (TLR9 agonist) [68,76].

##### TLR3 Agonist

Polyinosinic–polycytidylic acid (Poly(I:C)) is a synthetic double-stranded RNA (dsRNA) that mimics viral RNA, activating viral-infection-associated receptors like TLR3 and MDA5. This activation leads to the production of interferon and other cytokines, thereby stimulating the immune system [77,78]. NAKAO R et al. [66] demonstrated that the intranasal vaccination of mice with OMVs induced the production of Pg-specific antibodies in blood and saliva. Adding Poly(I:C) significantly boosted antibody levels, remarkably increasing serum IgG and salivary sIgA levels. Derivatives such as Poly-ICLC, Poly-IC12U, and PIKA Adjuvant (PIKA) offer improved stability, safety, and resistance to RNA enzymes and have been utilized in various vaccine clinical trials [68,79].

##### TLR4 Agonist

LPS binds to TLR4, causing conformational changes that activate downstream signaling pathways, including MyD88 and TRIF, leading to cellular inflammatory and immune responses [80,81]. MPL, a low-toxicity derivative of LPS, is commonly used in formulations like AS01 and AS04 [82]. AS01 is utilized in the malaria vaccine RTS, while S [83] and AS04 are utilized in vaccines like Cervarix™ and Fendrix™ [84,85]. Yang et al. used rHagB + MPL for nasal immunization in mice, resulting in significantly higher levels of specific antibodies than rHagB alone [36].

##### TLR5 Agonist

Flagellin (bacterial flagellin, FlaB) is a critical component of the bacterial flagellum that triggers the production of tumor necrosis factor-alpha (TNF-α) through TLR5. Combined with vaccine antigens, it leads to high antibody titers and a mixed Th1/Th2 response [86,87]. Puth et al. utilized a peptide from the Hgp44 structural domain of RgpA as a mucosal antigen, administering it to mice intranasally and sublingually with FlaB as an adjuvant [88]. This resulted in elevated levels of serum IgG and salivary sIgA antibodies compared to immunization with the antigen alone. Furthermore, a combination of Hgp44 and FlaB provided protection against alveolar bone loss triggered by Pg infection in mice. Intranasal immunization with the Hgp44-FlaB fusion protein generated similar levels of Hgp44-specific antibodies as Hgp44 + FlaB [88]. Recent research has explored the use of de-immunogenic dFlaB adjuvants, which have shown enhanced antigen-specific immune responses in both systemic and mucosal compartments without inducing FlaB-specific antibodies [89]. The development of de-immunogenic FlaB holds promise for broader clinical applications.

##### TLR9 Agonist

TLR9 is expressed on human plasmacytoid dendritic cells (DCs) and B cells, recognizing bacterial and viral DNA to trigger an innate immune response characterized by the production of Th1 and pro-inflammatory cytokines [90,91]. CpG oligodeoxynucleotides (CpG-ODN) are synthetic molecules designed to stimulate TLR9 by mimicking bacterial DNA at CpG sites, activating the natural immune system through this pathway. Studies by Chang et al. utilized rGroEL as an antigen and CpG-ODN as an adjuvant for the sublingual immunization of BALB/c mice, showing that CpG-ODN significantly increased levels of rGroEL-specific serum IgG and sIgA antibodies, suggesting that this strategy could be effective in preventing periodontal disease by inducing specific antibodies in mucosal and systemic systems [12]. Additionally, research by Liu et al. [92] using OMP and Bai et al. [14] using a periodontitis gene vaccine (pVAX1-HA2-fimA) demonstrated that CpG-ODN effectively induced a mucosal sIgA response, inhibited inflammation, and reduced bone loss. Furthermore, CpG-ODN has been shown to enhance vaccine immunization, with the FDA approving the first HEPLISAV-B™ Hepatitis B vaccine with CpG-ODN as an adjuvant in 2017 [93].

#### 3.1.3. FMS-like Tyrosine Kinase 3 Ligand

FMS-like tyrosine kinase 3 ligand (FLT3L) plays a crucial role in the proliferation and differentiation of early hematopoietic precursor stem cells in both human and mouse models. Additionally, it has been shown to stimulate the proliferation and differentiation of DCs [94], thereby enhancing antigen presentation and immune response. In a study by Zhang et al., using an OMP with a cDNA vector plasmid encoding FLT3 ligand (pFL) administered sublingually to mice resulted in a significant increase in serum IgG, IgA, and salivary sIgA antibodies, comparable to levels induced by OMP combined with a CT adjuvant [56]. Furthermore, mice immunized with OMP and pFL sublingually showed a notable reduction in alveolar bone loss [56]. In a separate study by Kobuchi et al., a dual adjuvant composed of pFL and CpG-ODN was used to nasal immunize mice with rFimA as the immunogen [13]. This led to a DC-mediated rise in rFimA-specific sIgA antibodies, indicating a potential role for these antibodies in preventing the binding of Pg to salivary-rich casein.

#### 3.1.4. Cytokines

Cytokines, a class of proteins or glycoproteins secreted by specific cells, play crucial roles in regulating immune responses and promoting cell functions such as proliferation, differentiation, and survival [95]. Recombinant analogs of cytokines may have limited adjuvant activity due to their short serum half-lives. To address this, cytokines often combine with liposomes or cytokine expression vectors for co-administration with DNA vaccines [96]. Currently, IL-15 is a common choice in Pg vaccines. Guo et al. [97] developed the co-expression plasmid pIRES-fimA:IL-15 and immunized mice via nasal or intramuscular routes. Their findings demonstrated that intranasal immunization enhanced antibody-specific immunity and systemic immune responses in the oral region, with IL-15 elevating FimA-specific sIgA antibody levels. Cytokine adjuvants represent a promising vaccine technology to enhance immunogenicity and protective efficacy, although further research is needed to address cost and safety concerns.

#### 3.1.5. Live Vectors

Live vector vaccines utilize natural or modified microorganisms to carry and deliver disease-related antigens [98]. These vectors can be bacterial or viral. *Streptococcus gordonii* (Sg), a human oral commensal, has been engineered to express the FimA antigen on its surface. Immunization with these recombinants in rats via oral administration resulted in the production of FimA-specific antibodies, offering protection against Pg-induced alveolar bone loss [99]. Similarly, a recombinant vaccine strain expressing HagA was developed using non-toxic *Salmonella typhimurium* as a vector. Mice inoculated with this strain produced antibodies, demonstrating successful delivery through the mucosal immune system [100]. Mice immunized orally with *Salmonella typhimurium* expressing HagB induced high levels of sIgA antibodies in saliva, with the extracellular HagB-expressing strain leading to higher levels of serum IgG and IgA antibodies [101]. Live vectors offer persistent stimulation of both humoral and cellular immunity, eliminating the need for target antigen purification, reducing vaccine dosage, and minimizing the required number of vaccinations.

#### 3.1.6. Saponin

Saponins, commonly found in plants, possess natural surfactant properties that enable them to bind vaccine antigens, forming antigen–saponin complexes that enhance the immunization efficacy of vaccines [67]. Acting as adjuvants, saponins can activate antigen-presenting cells (e.g., DCs) and T cells of the immune system, further boosting vaccine effectiveness [102,103]. Zhang et al. developed a vaccine using GPI-0100, a safer and more stable derivative of saponins, as an adjuvant, alongside HagB as an antigen [37]. Their study demonstrated that the subcutaneous injection of GPI-0100 led to higher vaccine immunization levels than MPL or alum, resulting in increased serum IgG antibodies. Similarly, intranasal immunization with GPI-0100 generated higher levels of serum and mucosal-specific HagB antibodies compared to MPL, alum, or CTB. While saponins offer advantages such as reducing the number of vaccinations required and prolonging the vaccine’s duration of action, they also present challenges like hemolysis and cytotoxicity. The new ISCOM^TM^ and ISCOMATRIX^TM^ vaccine formulations retain saponins’ adjuvant activity while addressing these concerns [104]. However, issues like bubble formation and pH sensitivity should be carefully considered when selecting and using saponin adjuvants to ensure their effectiveness and reliability.

**Table 1:** The adjuvants used in the Pg vaccine.

Adjuvant/Delivery	Antigen	Model	Administration	Ref.
CT	OMP	Mouse	in., oral, sl.	[20,21]
mCTA/LTB	OMP	Mouse	in.	[22]
CTB	FimA	Mouse	in., oral	[30,71]
	FimA (DNA)	Mouse	oral	[31]
	Kgp (HArep domain)	Mouse	in.	[72,105]
MPL	Kgp (HArep domain)	Mouse	in.	[105]
	rHagB	Mouse	in.	[36]
Poly (I:C)	OMV	Mouse	in.	[65,66]
FlaB	RgpA (Hgp44 domain)	Mouse	in., sl.	[88]
CpG-ODN	Fima/HA2 (DNA)	Rat	in.	[14]
	GroEL	Mouse	sl.	[12]
	OMP	Mouse	oral	[92]
DNA plasmid: CpG-ODN	FimA	Mouse	in.	[13]
DNA plasmid: pFL	OMP	Mouse	sl.	[56]
DNA plasmid: Flt3l	FimA	Mouse	in.	[13]
DNA plasmid: IL-15	FimA (DNA)	Mouse	in.	[97]
	FimA/HA2 (DNA)	Rat	in.	[14]
Live carrier: Streptococcus gordonii	FimA	Rat	oral	[99]
Live carrier: *Salmonella typhimurium*	HagA/B	Mouse	oral	[100,101]
saponin derivative GPI-0100	HagB	Mouse	sc., in.	[37]
Liposome GM-53 or MDP-Lys(L18)	FimA	Mouse	oral, sc.	[106,107]
HVJ envelope vector	RgpA (DNA)	Mouse	in.	[108]

CpG-ODN: CpG oligodeoxynucleotides; CT(B): cholera toxin (subunit B); FimA: Fimbriae; FimA/HA2: Fimbriae/Hemagglutinin-2; FlaB: a major flagellin of Vibrio vulnificus; Flt3L: FMS-like tyrosine kinase 3 ligand; GPI-0100: a fractionated quillaja saponin derivative; GroEL: a homolog of heat shock protein 60; (r)Hag(A/B): (recombinant) hemagglutinin (A/B); HVJ: hemagglutinating virus of Japan; IL-15: interleukin-15; in.: intranasal; Kgp: lysine gingipain; mCTA/LTB: mutant A subunit cholera toxin/B subunit heat-labile toxin; MPL: monophosporyl lipid A; OMP: outer membrane protein; OMV: outer membrane vesicles; pFL: plasmid containing the Flt3 ligand; poly (I:C): polyriboinosinic polyribocytidylic acid; RgpA: arginine-specific gingipain; sc.: subcutaneous; sl.: sublingual.

### 3.2. Antigen Delivery Adjuvants

#### 3.2.1. Liposomes

Liposomes are small spherical structures comprising lipid molecules like phospholipids and cholesterol, featuring hydrophobic and hydrophilic regions. These liposomes can effectively deliver antigens to immune cells in a targeted manner, enhance antigen cross-presentation, and stimulate the desired immune response [109]. Ogawa et al. conducted an experiment where mice were orally immunized with liposomes containing FimA and muscle-modulating factor 53 (GM-53), producing anti-FimA-specific IgG and IgA antibodies in the serum [106]. A significant increase in IgA-producing spot-forming cells was observed in the intestinal lamina propria and mesenteric lymph nodes, along with a rise in total Ig-producing cells [106]. Furthermore, co-mixing FimA with GM-53 or MDP-Lys (L18) in liposomes and immunizing mice through subcutaneous or oral routes led to elevated levels of serum anti-FimA IgG, IgA, and IgM antibodies, with IgG being the predominant type. Mice immunized orally exhibited notably higher salivary sIgA antibody levels against FimA than those immunized with FimA alone [107]. Over the years, various delivery systems incorporating liposomes have been developed and utilized in approved vaccines for diseases like malaria and herpes zoster [109].

#### 3.2.2. Lipid Nanoparticles

Lipid nanoparticles (LNPs) are microparticles composed of lipids with a wide range of applications, including drug delivery, gene therapy, and immunology. LNPs are utilized in vaccine development to package and deliver various antigens like proteins, carbohydrates, and nucleic acids [110]. Notably, COVID-19 vaccines mRNA-1273 and BNT162b2 utilize LNPs to deliver antigenic mRNAs [111]. LNPs exhibit good biocompatibility and biodegradability, making them suitable as mucosal vaccine adjuvants to improve vaccine adhesion, stability, immunogenicity, and protective effects. The use of LNPs as novel vaccine adjuvants in Pg vaccines shows promise, warranting further research to explore their application potential.

#### 3.2.3. Virus-like Particles

Virus-like particles (VLPs) are non-infectious particles that mimic intact viral particles in size and structure, formed through the self-assembly of viral capsid proteins without containing the viral genome [112]. These VLPs stimulate the immune system to generate an immune response similar to natural viral infections, ensuring high immunogenicity and safety. Miyachi et al. [108] demonstrated that using a gene gun abdominal immunization or the hemagglutinating virus of Japan (HVJ) nasal immunization with RgpA DNA vaccine resulted in the production of serum IgG and sIgA antibodies, with nasal immunization showing a stronger effect compared to abdominal immunization. While both methods could prevent alveolar bone loss, nasal immunization with HVJ envelope carrier proved more effective [108]. Additionally, VLPs have been explored for developing vaccines for tumors, AIDS, influenza, and other diseases [113].

#### 3.2.4. Microneedle Array

Microneedle arrays are composed of numerous micron-sized tips connected to a base in an array, piercing the skin surface to create small channels for the delivery of drugs or vaccines into deeper skin layers or mucous membranes [114,115]. Microneedle arrays were introduced for transdermal drug delivery research in the 1990s [116]; microneedle arrays come in various forms, including solid, coated, soluble, hollow, and hydrogel microneedles, along with the innovative cryo-microneedle array technology [117,118]. These arrays offer painless administration, improved vaccination compliance, and the potential for continuous dosing. Despite these advantages, challenges related to the efficiency and safety of microneedle delivery systems need to be addressed. Microneedles for DNA vaccine delivery to the skin [119,120] or mucosal tissues [121] have shown promise and may be explored for future applications, such as in Pg vaccines.

## 4. Immune Pathway

Vaccine immunization routes encompass intramuscular injection, subcutaneous injection, sublingual immunization, buccal mucosal immunization, nasal immunization, and oral immunization [122,123,124]. The choice of immunization route significantly impacts the immunization effects, highlighting the importance of selecting the appropriate route. Given that Pg resides in periodontal pockets, an effective Pg vaccine should trigger a robust protective antibody response in the oral mucosa. Consequently, most Pg vaccines are administered through mucosal routes. sIgA serves as the primary antibody in mucosal immune responses, capable of encapsulating organisms, neutralizing toxins, and inhibiting Pg adherence to mucosal and dental surfaces [13,125,126,127]. Therefore, enhancing the main antibody potency of sIgA is essential to improve the immunization efficacy of Pg vaccines.

### 4.1. Mucosal Immunity

Mucosal immunization offers several advantages, including ease of administration, potential for increased patient compliance, lower side effects, and enhanced immunity [69,128]. However, challenges exist, such as the susceptibility of oral mucosal vaccines to degradation in the gastrointestinal tract and the need for higher antigen doses to elicit the desired immune response [127,129]. In contrast, sublingual delivery avoids degradation by gastric juices and gastrointestinal enzymes [130], despite some enzymatic activity in the oral cavity. However, excessive secretions and salivary flow may dilute the antigen or lead to swallowing before mucosal absorption [131]. The vaginal mucosal route boasts low enzyme activity and a large surface area for antigen invasion, making it a potential candidate for self-administration [132]. Nasal immunization, on the other hand, triggers specific mucosal and systemic immune responses at lower antigen doses, thereby reducing the risk of anaphylaxis. Nasal vaccines demonstrate efficacy against not only infectious diseases but also non-infectious chronic conditions like obesity [133], hypertension [134] and type 2 diabetes [135]. This painless and readily accepted method effectively boosts mucosal immunity and stands out as a well-studied mode of immunization that enhances the effectiveness of Pg vaccines. Nevertheless, nasal vaccination faces challenges such as short nasal residence time, rapid antigen clearance and immune tolerance that need to be addressed [136].

### 4.2. The Application of Mucosal Immune Pathways in Pg Vaccine

Studies have indicated that both subcutaneous and intranasal immunization of mice with OMV produced IgG antibodies in serum [66]. However, the levels of sIgA produced in the saliva of mice immunized subcutaneously were similar to those of the PBS group [66]. Intramuscular injection, the most commonly used method for vaccine immunization, significantly increased the potency of serum IgG and IgA antibodies but not sIgA antibodies. In studies involving children, the FluMist, a live attenuated influenza vaccine, was administered via both nasal and intramuscular routes. The results demonstrated that nasal administration provided better protective immunity than intramuscular administration [137]. Therefore, intramuscular injection is not recommended as an appropriate method for Pg vaccines.

The mucosal route effectively achieves an immune response that induces higher levels of antibody production and prevents Pg-induced alveolar bone loss. Studies of mice showed that oral immunization led to the production of specific antibodies in serum, aiding the prevention of Pg infection [21,92,99,100]. These studies demonstrated successful delivery of the target protein through the intestinal mucosa. Additionally, various studies have highlighted the efficacy of nasal [13,20,22,138,139] or sublingual [12,21,34,56,88] immunization routes, resulting in high levels of serum-specific IgG, IgA, and salivary sIgA. These antibodies have proven effective in preventing Pg-induced infections and alveolar bone loss.

## 5. Status and Challenges of Pg Vaccine

### 5.1. Current Situation

Antigens for Pg vaccines, including FimA, Gingipains, and outer membrane proteins, are being extensively researched and evaluated. These antigens have shown promising potential in protecting against Pg infection. Inactivated, subunit, and DNA vaccines have been explored in preclinical studies. Subunit vaccines are favored over whole-cell inactivated vaccines to minimize the risk of adverse reactions due to multiple antigenic determinants. However, subunit vaccines may not offer sufficient immunity, leading to the incorporation of adjuvants in most Pg vaccine formulations to enhance immunity and ensure long-term protection. Different adjuvants like LT, CT, and TLR ligands have been investigated, along with using vectors (such as live bacterial and plasmid vectors) and delivery systems (like liposomes and virus-like particles) to enhance vaccine immunogenicity and stability. While most Pg vaccines are administered nasally or orally, recent advancements have explored sublingual or buccal mucosa delivery. Only a limited number of studies have investigated subcutaneous or intramuscular administration, which may be related to the location of the Pg infection and colonization. Mucosal immunization activates both local and systemic immune responses, whereas subcutaneous or intramuscular vaccination primarily induces systemic immune responses.

### 5.2. Challenges

The rapid advancement of Pg vaccines presents numerous challenges that must be addressed. One key hurdle is the complex and diverse strains of Pg, necessitating the selection of antigens capable of targeting multiple strains. Moreover, Pg exhibits highly variable surface antigens and adhesion factors, further complicating vaccine development. Ensuring the effectiveness and safety of the vaccine requires careful selection of antigens that provide long-term immune protection. While adjuvants have shown progress, there are still significant barriers to overcome. Live vectors raise concerns about genetic engineering modifications, while delivery systems such as liposomes or virus-like particles must precisely control antigen release efficiency and duration. The production process is intricate, with issues of instability and toxicity that demand resolution. Additionally, the choice of immunization route is crucial for enhancing vaccine efficacy. Typical routes for Pg vaccines include nasal, oral, and sublingual administration. Although these routes can enhance immunization, they also present challenges. Nasal immunization has dosage and residence time limitations in the nasal cavity, oral immunization risks antigen degradation, and sublingual immunization has issues with unstable saliva flushing that may impact vaccine effectiveness. It is crucial for a vaccine to address logistical challenges and meet the rigorous standards set by regulators, ensuring a balance between safety and effectiveness.

## 6. Conclusions

Periodontal disease is a widespread global issue, with Pg being a significant pathogen linked to this condition and other diseases. Despite the challenges involved in treating periodontal disease through conventional means, extensive research on Pg vaccines has shown their potential for entering clinical trials and gaining approval. Recent research has demonstrated cross-reactivity among various serotypes of Pg and between Pg and other oral pathogens [61,140,141]. This indicates the possibility of future vaccines offering cross-protection as universal vaccines to prevent a broad spectrum of oral pathogens. The diverse range of available adjuvants serves as a strong foundation for developing Pg vaccines. For instance, adjuvants like CT and LT improve the mucosal uptake of antigens, while CTB subunit vaccines have shown reliable safety profiles in human trials [69]. Additionally, vaccines incorporating CpG-OND as an adjuvant have received marketing approval [93,142], with all these adjuvants proving effective in the mucosal pathway, guiding the development of Pg vaccines. Furthermore, novel delivery systems such as chitosan nanoparticle carriers, microneedle arrays, and immunostimulatory complexes are being utilized. In conclusion, with extensive research, technological advancements, and clinical validation, our knowledge of Pg vaccines will continue to expand. This will lead to the accelerated development and implementation of Pg vaccines in clinical settings, offering effective protection against Pg-related diseases.

## Data Availability

Not applicable.

## References

[1] Xu W., Zhou W., Wang H., Liang S. (2020). Roles of *Porphyromonas gingivalis* and its virulence factors in periodontitis. Adv. Protein Chem. Struct. Biol..

[2] Lunar Silva I., Cascales E. (2021). Molecular Strategies Underlying *Porphyromonas gingivalis* Virulence. J. Mol. Biol..

[3] Badiger A.B., Gowda T.M., Chandra K., Mehta D.S. (2019). Bilateral Interrelationship of Diabetes and Periodontium. Curr. Diabetes Rev..

[4] Lalla E., Papapanou P.N. (2011). Diabetes mellitus and periodontitis: A tale of two common interrelated diseases. Nat. Rev. Endocrinol..

[5] Sanz M., Marco Del Castillo A., Jepsen S., Gonzalez-Juanatey J.R., D’Aiuto F., Bouchard P., Chapple I., Dietrich T., Gotsman I., Graziani F. (2020). Periodontitis and cardiovascular diseases: Consensus report. J. Clin. Periodontol..

[6] Kavarthapu A., Gurumoorthy K. (2021). Linking chronic periodontitis and oral cancer: A review. Oral Oncol..

[7] Gao S., Li S., Ma Z., Liang S., Shan T., Zhang M., Zhu X., Zhang P., Liu G., Zhou F. (2016). Presence of *Porphyromonas gingivalis* in esophagus and its association with the clinicopathological characteristics and survival in patients with esophageal cancer. Infect. Agent. Cancer.

[8] Ogrendik M. (2023). The Association Between Oral Anaerobic Bacteria and Pancreatic Cancer. World J. Oncol..

[9] Tanaka S., Murakami Y., Ogiwara T., Shoji M., Seto K., Nagasaki M., Fujisawa S. (2002). Frequency of reactivity for *Porphyromonas gingivalis* and *Prevotella* spp. in supra- and subgingival plaques, and periodontal clinical parameters according to subject age. J. Periodontol..

[10] Ahmadi H., Ebrahimi A., Ahmadi F. (2021). Antibiotic Therapy in Dentistry. Int. J. Dent..

[11] Jepsen K., Falk W., Brune F., Fimmers R., Jepsen S., Bekeredjian-Ding I. (2021). Prevalence and antibiotic susceptibility trends of periodontal pathogens in the subgingival microbiota of German periodontitis patients: A retrospective surveillance study. J. Clin. Periodontol..

[12] Chang E., Kobayashi R., Hagiwara-Hamano M., Kurita-Ochiai T., Komiya M. (2022). Sublingual immunization with recombinant GroEL plus CpG-ODN inhibits *Porphyromonas gingivalis*-induced inflammation and alveolar bone loss. Mol. Oral Microbiol..

[13] Kobuchi K., Kataoka K., Taguchi Y., Miyake T., Umeda M. (2019). Nasal double DNA adjuvant induces salivary FimA-specific secretory IgA antibodies in young and aging mice and blocks *Porphyromonas gingivalis* binding to a salivary protein. BMC Oral Health.

[14] Bai G., Yu H., Guan X., Zeng F., Liu X., Chen B., Liu J., Tian Y. (2021). CpG immunostimulatory oligodeoxynucleotide 1826 as a novel nasal ODN adjuvant enhanced the protective efficacy of the periodontitis gene vaccine in a periodontitis model in SD rats. BMC Oral Health.

[15] Takahashi Y., Cueno M.E., Kamio N., Iinuma T., Hasegawa Y., Imai K. (2022). *Porphyromonas gingivalis* Mfa1 fimbria putatively binds to TLR2 and induces both IL-6 and IL-8 production in human bronchial epithelial cells. Biochem. Biophys. Res. Commun..

[16] Miya C., Cueno M.E., Suzuki R., Maruoka S., Gon Y., Kaneko T., Yonehara Y., Imai K. (2021). *Porphyromonas gingivalis* gingipains potentially affect MUC5AC gene expression and protein levels in respiratory epithelial cells. FEBS Open Bio.

[17] Rocha F.G., Berges A., Sedra A., Ghods S., Kapoor N., Pill L., Davey M.E., Fairman J., Gibson F.C. (2021). A *Porphyromonas gingivalis* Capsule-Conjugate Vaccine Protects From Experimental Oral Bone Loss. Front. Oral Health.

[18] Gonzalez D., Tzianabos A.O., Genco C.A., Gibson F.C. (2003). Immunization with *Porphyromonas gingivalis* capsular polysaccharide prevents *P. gingivalis*-elicited oral bone loss in a murine model. Infect. Immun..

[19] Maeba S., Otake S., Namikoshi J., Shibata Y., Hayakawa M., Abiko Y., Yamamoto M. (2005). Transcutaneous immunization with a 40-kDa outer membrane protein of *Porphyromonas gingivalis* induces specific antibodies which inhibit coaggregation by *P. gingivalis*. Vaccine.

[20] Cai Y., Kurita-Ochiai T., Kobayashi R., Hashizume T., Yamamoto M. (2013). Nasal immunization with the 40-kDa outer membrane protein of *Porphyromonas gingivalis* plus cholera toxin induces protective immunity in aged mice. J. Oral Sci..

[21] Ikeda T., Kobayashi R., Kurita-Ochiai T. (2014). Comparison of mucosal immune response after oral, nasal or sublingual immunization with an outer membrane protein of *Porphyromonas gingivalis*. Int. J. Oral-Med. Sci..

[22] Momoi F., Hashizume T., Kurita-Ochiai T., Yuki Y., Kiyono H., Yamamoto M. (2008). Nasal vaccination with the 40-kilodalton outer membrane protein of *Porphyromonas gingivalis* and a nontoxic chimeric enterotoxin adjuvant induces long-term protective immunity with reduced levels of immunoglobulin E antibodies. Infect. Immun..

[23] Fujiwara-Takahashi K., Watanabe T., Shimogishi M., Shibasaki M., Umeda M., Izumi Y., Nakagawa I. (2020). Phylogenetic diversity in fim and mfa gene clusters between *Porphyromonas gingivalis* and *Porphyromonas gulae*, as a potential cause of host specificity. J. Oral Microbiol..

[24] Yoshimura F., Murakami Y., Nishikawa K., Hasegawa Y., Kawaminami S. (2009). Surface components of *Porphyromonas gingivalis*. J. Periodontal Res..

[25] Kugaji M., Muddapur U., Bhat K., Joshi V., Manubolu M., Pathakoti K., Peram M.R., Kumbar V. (2020). Variation in the Occurrence of fimA Genotypes of *Porphyromonas gingivalis* in Periodontal Health and Disease. Int. J. Environ. Res. Public Health.

[26] Wang Q., Zhou X.D., Zheng Q.H., Wang Y., Tang L., Huang D.M. (2010). Distribution of *Porphyromonas gingivalis* fimA genotypes in chronic apical periodontitis associated with symptoms. J. Endod..

[27] Moreno S., Contreras A. (2013). Functional differences of *Porphyromonas gingivalis* Fimbriae in determining periodontal disease pathogenesis: A literature review. Colomb. Med..

[28] Hasegawa Y., Nagano K. (2021). *Porphyromonas gingivalis* FimA and Mfa1 fimbriae: Current insights on localization, function, biogenesis, and genotype. Jpn. Dent. Sci. Rev..

[29] Koh E.M., Kim J., Kim T.G., Moon J.H., Oh J.H., Lee J.Y., Jang Y.S. (2011). Cloning and characterization of heavy and light chain genes encoding the FimA-specific monoclonal antibodies that inhibit *Porphyromonas gingivalis* adhesion. Microbiol. Immunol..

[30] Takahashi Y., Kumada H., Hamada N., Haishima Y., Ozono S., Isaka M., Yasuda Y., Tochikubo K., Umemoto T. (2007). Induction of immune responses and prevention of alveolar bone loss by intranasal administration of mice with *Porphyromonas gingivalis* fimbriae and recombinant cholera toxin B subunit. Oral Microbiol. Immunol..

[31] Shin E.A., Lee J.Y., Kim T.G., Park Y.K., Langridge W.H. (2006). Synthesis and assembly of an adjuvanted *Porphyromonas gingivalis* fimbrial antigen fusion protein in plants. Protein Expr. Purif..

[32] Aleksijević L.H., Aleksijević M., Škrlec I., Šram M., Šram M., Talapko J. (2022). *Porphyromonas gingivalis* Virulence Factors and Clinical Significance in Periodontal Disease and Coronary Artery Diseases. Pathogens.

[33] Smalley J.W., Olczak T. (2017). Heme acquisition mechanisms of *Porphyromonas gingivalis*—Strategies used in a polymicrobial community in a heme-limited host environment. Mol. Oral Microbiol..

[34] Yuzawa S., Kurita-Ochiai T., Hashizume T., Kobayashi R., Abiko Y., Yamamoto M. (2012). Sublingual vaccination with fusion protein consisting of the functional domain of hemagglutinin A of *Porphyromonas gingivalis* and *Escherichia coli* maltose-binding protein elicits protective immunity in the oral cavity. FEMS Immunol. Med. Microbiol..

[35] Katz J., Black K.P., Michalek S.M. (1999). Host responses to recombinant hemagglutinin B of *Porphyromonas gingivalis* in an experimental rat model. Infect. Immun..

[36] Yang Q.B., Martin M., Michalek S.M., Katz J. (2002). Mechanisms of monophosphoryl lipid A augmentation of host responses to recombinant HagB from *Porphyromonas gingivalis*. Infect. Immun..

[37] Zhang P., Yang Q.B., Marciani D.J., Martin M., Clements J.D., Michalek S.M., Katz J. (2003). Effectiveness of the quillaja saponin semi-synthetic analog GPI-0100 in potentiating mucosal and systemic responses to recombinant HagB from *Porphyromonas gingivalis*. Vaccine.

[38] Gaddis D.E., Michalek S.M., Katz J. (2009). Requirement of TLR4 and CD14 in dendritic cell activation by Hemagglutinin B from *Porphyromonas gingivalis*. Mol. Immunol..

[39] Guo Y., Nguyen K.A., Potempa J. (2010). Dichotomy of gingipains action as virulence factors: From cleaving substrates with the precision of a surgeon’s knife to a meat chopper-like brutal degradation of proteins. Periodontol. 2000.

[40] de Jongh C.A., Bikker F.J., de Vries T.J., Werner A., Gibbs S., Krom B.P. (2024). *Porphyromonas gingivalis* interaction with Candida albicans allows for aerobic escape, virulence and adherence. Biofilm.

[41] Olsen I., Potempa J. (2014). Strategies for the inhibition of gingipains for the potential treatment of periodontitis and associated systemic diseases. J. Oral Microbiol..

[42] Chow Y.C., Yam H.C., Gunasekaran B., Lai W.Y., Wo W.Y., Agarwal T., Ong Y.Y., Cheong S.L., Tan S.A. (2022). Implications of *Porphyromonas gingivalis* peptidyl arginine deiminase and gingipain R in human health and diseases. Front. Cell Infect. Microbiol..

[43] Gibson F.C., Genco C.A. (2001). Prevention of *Porphyromonas gingivalis*-induced oral bone loss following immunization with gingipain R1. Infect. Immun..

[44] Mishra A., Roy F., Dou Y., Zhang K., Tang H., Fletcher H.M. (2018). Role of Acetyltransferase PG1842 in Gingipain Biogenesis in *Porphyromonas gingivalis*. J. Bacteriol..

[45] Wilensky A., Potempa J., Houri-Haddad Y., Shapira L. (2017). Vaccination with recombinant RgpA peptide protects against *Porphyromonas gingivalis*-induced bone loss. J. Periodontal Res..

[46] O’Brien-Simpson N.M., Pathirana R.D., Paolini R.A., Chen Y.Y., Veith P.D., Tam V., Ally N., Pike R.N., Reynolds E.C. (2005). An immune response directed to proteinase and adhesin functional epitopes protects against *Porphyromonas gingivalis*-induced periodontal bone loss. J. Immunol..

[47] Frazer L.T., O’Brien-Simpson N.M., Slakeski N., Walsh K.A., Veith P.D., Chen C.G., Barr I.G., Reynolds E.C. (2006). Vaccination with recombinant adhesins from the RgpA-Kgp proteinase-adhesin complex protects against *Porphyromonas gingivalis* infection. Vaccine.

[48] van Winkelhoff A.J., Appelmelk B.J., Kippuw N., de Graaff J. (1993). K-antigens in *Porphyromonas gingivalis* are associated with virulence. Oral Microbiol. Immunol..

[49] Laine M.L., Appelmelk B.J., van Winkelhoff A.J. (1996). Novel polysaccharide capsular serotypes in *Porphyromonas gingivalis*. J. Periodontal Res..

[50] d’Empaire G., Baer M.T., Gibson F.C. (2006). The K1 serotype capsular polysaccharide of *Porphyromonas gingivalis* elicits chemokine production from murine macrophages that facilitates cell migration. Infect. Immun..

[51] Laine M.L., Appelmelk B.J., van Winkelhoff A.J. (1997). Prevalence and distribution of six capsular serotypes of *Porphyromonas gingivalis* in periodontitis patients. J. Dent. Res..

[52] Schifferle R.E., Chen P.B., Davern L.B., Aguirre A., Genco R.J., Levine M.J. (1993). Modification of experimental *Porphyromonas gingivalis* murine infection by immunization with a polysaccharide-protein conjugate. Oral Microbiol. Immunol..

[53] Veith P.D., Gorasia D.G., Reynolds E.C. (2021). Towards defining the outer membrane proteome of *Porphyromonas gingivalis*. Mol. Oral Microbiol..

[54] Hamada N., Watanabe K., Tahara T., Nakazawa K., Ishida I., Shibata Y., Kobayashi T., Yoshie H., Abiko Y., Umemoto T. (2007). The r40-kDa outer membrane protein human monoclonal antibody protects against *Porphyromonas gingivalis*-induced bone loss in rats. J. Periodontol..

[55] Namikoshi J., Otake S., Maeba S., Hayakawa M., Abiko Y., Yamamoto M. (2003). Specific antibodies induced by nasally administered 40-kDa outer membrane protein of *Porphyromonas gingivalis* inhibits coaggregation activity of *P. gingivalis*. Vaccine.

[56] Zhang T., Hashizume T., Kurita-Ochiai T., Yamamoto M. (2009). Sublingual vaccination with outer membrane protein of *Porphyromonas gingivalis* and Flt3 ligand elicits protective immunity in the oral cavity. Biochem. Biophys. Res. Commun..

[57] Romero-Lastra P., Sánchez M.C., Llama-Palacios A., Figuero E., Herrera D., Sanz M. (2019). Gene expression of *Porphyromonas gingivalis* ATCC 33277 when growing in an in vitro multispecies biofilm. PLoS ONE.

[58] Fröhlich E., Kantyka T., Plaza K., Schmidt K.H., Pfister W., Potempa J., Eick S. (2013). Benzamidine derivatives inhibit the virulence of *Porphyromonas gingivalis*. Mol. Oral Microbiol..

[59] Hinode D., Grenier D., Mayrand D. (1995). Purification and characterization of a DnaK-like and a GroEL-like protein from *Porphyromonas gingivalis*. Anaerobe.

[60] Hinode D., Nakamura R., Grenier D., Mayrand D. (1998). Cross-reactivity of specific antibodies directed to heat shock proteins from periodontopathogenic bacteria and of human origin [corrected]. Oral Microbiol. Immunol..

[61] Choi J.I., Choi K.S., Yi N.N., Kim U.S., Choi J.S., Kim S.J. (2005). Recognition and phagocytosis of multiple periodontopathogenic bacteria by anti-*Porphyromonas gingivalis* heat-shock protein 60 antisera. Oral Microbiol. Immunol..

[62] Lee J.Y., Yi N.N., Kim U.S., Choi J.S., Kim S.J., Choi J.I. (2006). *Porphyromonas gingivalis* heat shock protein vaccine reduces the alveolar bone loss induced by multiple periodontopathogenic bacteria. J. Periodontal Res..

[63] Deng D.K., Zhang J.J., Gan D., Zou J.K., Wu R.X., Tian Y., Yin Y., Li X., Chen F.M., He X.T. (2022). Roles of extracellular vesicles in periodontal homeostasis and their therapeutic potential. J. Nanobiotechnol..

[64] Okamura H., Hirota K., Yoshida K., Weng Y., He Y., Shiotsu N., Ikegame M., Uchida-Fukuhara Y., Tanai A., Guo J. (2021). Outer membrane vesicles of *Porphyromonas gingivalis*: Novel communication tool and strategy. Jpn. Dent. Sci. Rev..

[65] Nakao R., Hasegawa H., Ochiai K., Takashiba S., Ainai A., Ohnishi M., Watanabe H., Senpuku H. (2011). Outer membrane vesicles of *Porphyromonas gingivalis* elicit a mucosal immune response. PLoS ONE.

[66] Nakao R., Hasegawa H., Dongying B., Ohnishi M., Senpuku H. (2016). Assessment of outer membrane vesicles of periodontopathic bacterium *Porphyromonas gingivalis* as possible mucosal immunogen. Vaccine.

[67] Gao Y., Guo Y. (2023). Research progress in the development of natural-product-based mucosal vaccine adjuvants. Front. Immunol..

[68] Firdaus F.Z., Skwarczynski M., Toth I. (2022). Developments in Vaccine Adjuvants. Methods Mol. Biol..

[69] Lavelle E.C., Ward R.W. (2022). Mucosal vaccines—Fortifying the frontiers. Nat. Rev. Immunol..

[70] Montero D.A., Vidal R.M., Velasco J., George S., Lucero Y., Gómez L.A., Carreño L.J., García-Betancourt R., O’Ryan M. (2023). Vibrio cholerae, classification, pathogenesis, immune response, and trends in vaccine development. Front. Med..

[71] Kim T.G., Huy N.X., Kim M.Y., Jeong D.K., Jang Y.S., Yang M.S., Langridge W.H., Lee J.Y. (2009). Immunogenicity of a cholera toxin B subunit *Porphyromonas gingivalis* fimbrial antigen fusion protein expressed in *E. coli*. Mol. Biotechnol..

[72] Zhang P., Yang Q.B., Balkovetz D.F., Lewis J.P., Clements J.D., Michalek S.M., Katz J. (2005). Effectiveness of the B subunit of cholera toxin in potentiating immune responses to the recombinant hemagglutinin/adhesin domain of the gingipain Kgp from *Porphyromonas gingivalis*. Vaccine.

[73] Qadri F., Akhtar M., Bhuiyan T.R., Chowdhury M.I., Ahmed T., Rafique T.A., Khan A., Rahman S.I.A., Khanam F., Lundgren A. (2020). Safety and immunogenicity of the oral, inactivated, enterotoxigenic *Escherichia coli* vaccine ETVAX in Bangladeshi children and infants: A double-blind, randomised, placebo-controlled phase 1/2 trial. Lancet Infect. Dis..

[74] Akhtar M., Chowdhury M.I., Bhuiyan T.R., Kaim J., Ahmed T., Rafique T.A., Khan A., Rahman S.I.A., Khanam F., Begum Y.A. (2019). Evaluation of the safety and immunogenicity of the oral inactivated multivalent enterotoxigenic *Escherichia coli* vaccine ETVAX in Bangladeshi adults in a double-blind, randomized, placebo-controlled Phase I trial using electrochemiluminescence and ELISA assays for immunogenicity analyses. Vaccine.

[75] Kaur A., Baldwin J., Brar D., Salunke D.B., Petrovsky N. (2022). Toll-like receptor (TLR) agonists as a driving force behind next-generation vaccine adjuvants and cancer therapeutics. Curr. Opin. Chem. Biol..

[76] Luchner M., Reinke S., Milicic A. (2021). TLR Agonists as Vaccine Adjuvants Targeting Cancer and Infectious Diseases. Pharmaceutics.

[77] Lamoot A., Jangra S., Laghlali G., Warang P., Singh G., Chang L.A., Park S.C., Singh G., De Swarte K., Zhong Z. (2023). Lipid Nanoparticle Encapsulation Empowers Poly(I:C) to Activate Cytoplasmic RLRs and Thereby Increases Its Adjuvanticity. Small.

[78] Ko K.H., Cha S.B., Lee S.H., Bae H.S., Ham C.S., Lee M.G., Kim D.H., Han S.H. (2023). A novel defined TLR3 agonist as an effective vaccine adjuvant. Front. Immunol..

[79] Stahl-Hennig C., Eisenblätter M., Jasny E., Rzehak T., Tenner-Racz K., Trumpfheller C., Salazar A.M., Uberla K., Nieto K., Kleinschmidt J. (2009). Synthetic double-stranded RNAs are adjuvants for the induction of T helper 1 and humoral immune responses to human papillomavirus in rhesus macaques. PLoS Pathog..

[80] Mata-Haro V., Cekic C., Martin M., Chilton P.M., Casella C.R., Mitchell T.C. (2007). The vaccine adjuvant monophosphoryl lipid A as a TRIF-biased agonist of TLR4. Science.

[81] Heine H., Zamyatina A. (2022). Therapeutic Targeting of TLR4 for Inflammation, Infection, and Cancer: A Perspective for Disaccharide Lipid A Mimetics. Pharmaceuticals.

[82] Duthie M.S., Windish H.P., Fox C.B., Reed S.G. (2011). Use of defined TLR ligands as adjuvants within human vaccines. Immunol. Rev..

[83] Coccia M., Collignon C., Hervé C., Chalon A., Welsby I., Detienne S., van Helden M.J., Dutta S., Genito C.J., Waters N.C. (2017). Cellular and molecular synergy in AS01-adjuvanted vaccines results in an early IFNγ response promoting vaccine immunogenicity. npj Vaccines.

[84] Haghshenas M.R., Mousavi T., Kheradmand M., Afshari M., Moosazadeh M. (2017). Efficacy of Human Papillomavirus L1 Protein Vaccines (Cervarix and Gardasil) in Reducing the Risk of Cervical Intraepithelial Neoplasia: A Meta-analysis. Int. J. Prev. Med..

[85] Fabrizi F., Cerutti R., Nardelli L., Tripodi F., Messa P. (2020). HBV vaccination with Fendrix is effective and safe in pre-dialysis CKD population. Clin. Res. Hepatol. Gastroenterol..

[86] Khim K., Puth S., Radhakrishnan K., Nguyen T.D., Lee Y.S., Jung C.H., Lee S.E., Rhee J.H. (2023). Deglycosylation of eukaryotic-expressed flagellin restores adjuvanticity. npj Vaccines.

[87] Clasen S.J., Bell M.E.W., Borbón A., Lee D.H., Henseler Z.M., de la Cuesta-Zuluaga J., Parys K., Zou J., Wang Y., Altmannova V. (2023). Silent recognition of flagellins from human gut commensal bacteria by Toll-like receptor 5. Sci. Immunol..

[88] Puth S., Hong S.H., Park M.J., Lee H.H., Lee Y.S., Jeong K., Kang I.C., Koh J.T., Moon B., Park S.C. (2017). Mucosal immunization with a flagellin-adjuvanted Hgp44 vaccine enhances protective immune responses in a murine *Porphyromonas gingivalis* infection model. Hum. Vaccin. Immunother..

[89] Khim K., Bang Y.J., Puth S., Choi Y., Lee Y.S., Jeong K., Lee S.E., Rhee J.H. (2021). Deimmunization of flagellin for repeated administration as a vaccine adjuvant. npj Vaccines.

[90] Kayraklioglu N., Horuluoglu B., Klinman D.M. (2021). CpG Oligonucleotides as Vaccine Adjuvants. Methods Mol. Biol..

[91] Iho S., Maeyama J., Suzuki F. (2015). CpG oligodeoxynucleotides as mucosal adjuvants. Hum. Vaccin. Immunother..

[92] Liu C., Hashizume T., Kurita-Ochiai T., Fujihashi K., Yamamoto M. (2010). Oral immunization with *Porphyromonas gingivalis* outer membrane protein and CpGoligodeoxynucleotides elicits T helper 1 and 2 cytokines for enhanced protective immunity. Mol. Oral Microbiol..

[93] Campbell J.D. (2017). Development of the CpG Adjuvant 1018: A Case Study. Methods Mol. Biol..

[94] Carlson K.N., Verhagen J.C., Jennings H., Verhoven B., McMorrow S., Pavan-Guimaraes J., Chlebeck P., Al-Adra D.P. (2022). Single-cell RNA sequencing distinguishes dendritic cell subsets in the rat, allowing advanced characterization of the effects of FMS-like tyrosine kinase 3 ligand. Scand. J. Immunol..

[95] Rahman T., Das A., Abir M.H., Nafiz I.H., Mahmud A.R., Sarker M.R., Emran T.B., Hassan M.M. (2023). Cytokines and their role as immunotherapeutics and vaccine Adjuvants: The emerging concepts. Cytokine.

[96] Tovey M.G., Lallemand C. (2010). Adjuvant activity of cytokines. Methods Mol. Biol..

[97] Guo H., Wang X., Jiang G., Yang P. (2006). Construction of a sIgA-enhancing anti-*Porphyromonas gingivalis* FimA vaccine and nasal immunization in mice. Immunol. Lett..

[98] Ding C., Ma J., Dong Q., Liu Q. (2018). Live bacterial vaccine vector and delivery strategies of heterologous antigen: A review. Immunol. Lett..

[99] Sharma A., Honma K., Evans R.T., Hruby D.E., Genco R.J. (2001). Oral immunization with recombinant Streptococcus gordonii expressing porphyromonas gingivalis FimA domains. Infect. Immun..

[100] Kozarov E., Miyashita N., Burks J., Cerveny K., Brown T.A., McArthur W.P., Progulske-Fox A. (2000). Expression and immunogenicity of hemagglutinin A from *Porphyromonas gingivalis* in an avirulent *Salmonella enterica* serovar typhimurium vaccine strain. Infect. Immun..

[101] Isoda R., Simanski S.P., Pathangey L., Stone A.E., Brown T.A. (2007). Expression of a *Porphyromonas gingivalis* hemagglutinin on the surface of a Salmonella vaccine vector. Vaccine.

[102] Chen K., Wang N., Zhang X., Wang M., Liu Y., Shi Y. (2023). Potentials of saponins-based adjuvants for nasal vaccines. Front. Immunol..

[103] Lee W., Suresh M. (2022). Vaccine adjuvants to engage the cross-presentation pathway. Front. Immunol..

[104] Skene C.D., Sutton P. (2006). Saponin-adjuvanted particulate vaccines for clinical use. Methods.

[105] Zhang P., Lewis J.P., Michalek S.M., Katz J. (2007). Role of CD80 and CD86 in host immune responses to the recombinant hemagglutinin domain of *Porphyromonas gingivalis* gingipain and in the adjuvanticity of cholera toxin B and monophosphoryl lipid A. Vaccine.

[106] Ogawa T., Kusumoto Y., Kiyono H., McGhee J.R., Hamada S. (1992). Occurrence of antigen-specific B cells following oral or parenteral immunization with *Porphyromonas gingivalis* fimbriae. Int. Immunol..

[107] Ogawa T., Shimauchi H., Hamada S. (1989). Mucosal and systemic immune responses in BALB/c mice to Bacteroides gingivalis fimbriae administered orally. Infect. Immun..

[108] Miyachi K., Ishihara K., Kimizuka R., Okuda K. (2007). Arg-gingipain A DNA vaccine prevents alveolar bone loss in mice. J. Dent. Res..

[109] Wang N., Chen M., Wang T. (2019). Liposomes used as a vaccine adjuvant-delivery system: From basics to clinical immunization. J. Control. Release.

[110] Chatzikleanthous D., O’Hagan D.T., Adamo R. (2021). Lipid-Based Nanoparticles for Delivery of Vaccine Adjuvants and Antigens: Toward Multicomponent Vaccines. Mol. Pharm..

[111] Hou X., Zaks T., Langer R., Dong Y. (2021). Lipid nanoparticles for mRNA delivery. Nat. Rev. Mater..

[112] Nooraei S., Bahrulolum H., Hoseini Z.S., Katalani C., Hajizade A., Easton A.J., Ahmadian G. (2021). Virus-like particles: Preparation, immunogenicity and their roles as nanovaccines and drug nanocarriers. J. Nanobiotechnol..

[113] Apostólico Jde S., Lunardelli V.A., Coirada F.C., Boscardin S.B., Rosa D.S. (2016). Adjuvants: Classification, Modus Operandi, and Licensing. J. Immunol. Res..

[114] Mdanda S., Ubanako P., Kondiah P.P.D., Kumar P., Choonara Y.E. (2021). Recent Advances in Microneedle Platforms for Transdermal Drug Delivery Technologies. Polymers.

[115] Creighton R.L., Woodrow K.A. (2019). Microneedle-Mediated Vaccine Delivery to the Oral Mucosa. Adv. Healthc. Mater..

[116] Henry S., McAllister D.V., Allen M.G., Prausnitz M.R. (1998). Microfabricated microneedles: A novel approach to transdermal drug delivery. J. Pharm. Sci..

[117] Waghule T., Singhvi G., Dubey S.K., Pandey M.M., Gupta G., Singh M., Dua K. (2019). Microneedles: A smart approach and increasing potential for transdermal drug delivery system. Biomed. Pharmacother..

[118] Chang H., Chew S.W.T., Zheng M., Lio D.C.S., Wiraja C., Mei Y., Ning X., Cui M., Than A., Shi P. (2021). Cryomicroneedles for transdermal cell delivery. Nat. Biomed. Eng..

[119] DeMuth P.C., Min Y., Huang B., Kramer J.A., Miller A.D., Barouch D.H., Hammond P.T., Irvine D.J. (2013). Polymer multilayer tattooing for enhanced DNA vaccination. Nat. Mater..

[120] Kim N.W., Lee M.S., Kim K.R., Lee J.E., Lee K., Park J.S., Matsumoto Y., Jo D.G., Lee H., Lee D.S. (2014). Polyplex-releasing microneedles for enhanced cutaneous delivery of DNA vaccine. J. Control. Release.

[121] Ma Y., Tao W., Krebs S.J., Sutton W.F., Haigwood N.L., Gill H.S. (2014). Vaccine delivery to the oral cavity using coated microneedles induces systemic and mucosal immunity. Pharm. Res..

[122] Trincado V., Gala R.P., Morales J.O. (2021). Buccal and Sublingual Vaccines: A Review on Oral Mucosal Immunization and Delivery Systems. Vaccines.

[123] Mokabari K., Iriti M., Varoni E.M. (2023). Mucoadhesive Vaccine Delivery Systems for the Oral Mucosa. J. Dent. Res..

[124] Zimmermann P., Curtis N. (2019). Factors That Influence the Immune Response to Vaccination. Clin. Microbiol. Rev..

[125] Patel H., Yewale C., Rathi M.N., Misra A. (2014). Mucosal immunization: A review of strategies and challenges. Crit. Rev. Ther. Drug Carrier Syst..

[126] Corthésy B. (2013). Multi-faceted functions of secretory IgA at mucosal surfaces. Front. Immunol..

[127] Saraf S., Jain S., Sahoo R.N., Mallick S. (2020). Present Scenario of M-Cell Targeting Ligands for Oral Mucosal Immunization. Curr. Drug Targets.

[128] Li M., Wang Y., Sun Y., Cui H., Zhu S.J., Qiu H.J. (2020). Mucosal vaccines: Strategies and challenges. Immunol. Lett..

[129] Shukla A., Khatri K., Gupta P.N., Goyal A.K., Mehta A., Vyas S.P. (2008). Oral immunization against hepatitis B using bile salt stabilized vesicles (bilosomes). J. Pharm. Pharm. Sci..

[130] Paris A.L., Colomb E., Verrier B., Anjuère F., Monge C. (2021). Sublingual vaccination and delivery systems. J. Control. Release.

[131] He S., Mu H. (2023). Microenvironmental pH Modification in Buccal/Sublingual Dosage Forms for Systemic Drug Delivery. Pharmaceutics.

[132] Singh J., Michel D., Getson H.M., Chitanda J.M., Verrall R.E., Badea I. (2015). Development of amino acid substituted gemini surfactant-based mucoadhesive gene delivery systems for potential use as noninvasive vaginal genetic vaccination. Nanomedicine.

[133] Azegami T., Yuki Y., Sawada S., Mejima M., Ishige K., Akiyoshi K., Itoh H., Kiyono H. (2017). Nanogel-based nasal ghrelin vaccine prevents obesity. Mucosal Immunol..

[134] Tissot A.C., Maurer P., Nussberger J., Sabat R., Pfister T., Ignatenko S., Volk H.D., Stocker H., Müller P., Jennings G.T. (2008). Effect of immunisation against angiotensin II with CYT006-AngQb on ambulatory blood pressure: A double-blind, randomised, placebo-controlled phase IIa study. Lancet.

[135] Pang Z., Nakagami H., Osako M.K., Koriyama H., Nakagami F., Tomioka H., Shimamura M., Kurinami H., Takami Y., Morishita R. (2014). Therapeutic vaccine against DPP4 improves glucose metabolism in mice. Proc. Natl. Acad. Sci. USA.

[136] Riese P., Sakthivel P., Trittel S., Guzmán C.A. (2014). Intranasal formulations: Promising strategy to deliver vaccines. Expert. Opin. Drug Deliv..

[137] Ashkenazi S., Vertruyen A., Arístegui J., Esposito S., McKeith D.D., Klemola T., Biolek J., Kühr J., Bujnowski T., Desgrandchamps D. (2006). Superior relative efficacy of live attenuated influenza vaccine compared with inactivated influenza vaccine in young children with recurrent respiratory tract infections. Pediatr. Infect. Dis. J..

[138] Sloat B.R., Cui Z. (2006). Nasal immunization with anthrax protective antigen protein adjuvanted with polyriboinosinic-polyribocytidylic acid induced strong mucosal and systemic immunities. Pharm. Res..

[139] Koizumi Y., Kurita-Ochiai T., Oguchi S., Yamamoto M. (2008). Nasal immunization with *Porphyromonas gingivalis* outer membrane protein decreases *P. gingivalis*-induced atherosclerosis and inflammation in spontaneously hyperlipidemic mice. Infect. Immun..

[140] Vasel D., Sims T.J., Bainbridge B., Houston L., Darveau R., Page R.C. (1996). Shared antigens of *Porphyromonas gingivalis* and *Bacteroides forsythus*. Oral Microbiol. Immunol..

[141] Fan Q., Sims T.J., Nakagawa T., Page R.C. (2000). Antigenic cross-reactivity among *Porphyromonas gingivalis* serotypes. Oral. Microbiol. Immunol..

[142] Hyer R.N., Janssen R.S. (2019). Immunogenicity and safety of a 2-dose hepatitis B vaccine, HBsAg/CpG 1018, in persons with diabetes mellitus aged 60–70 years. Vaccine.

